# Eravacycline and omadacycline Rescue multidrug-resistant *Elizabethkingia anophelis*: Triple-Model evaluation across *in Vitro*, *Galleria mellonella*, and murine bacteremia systems

**DOI:** 10.1016/j.jare.2025.11.048

**Published:** 2025-11-24

**Authors:** Shaohua Hu, Li Jiang, Hao Xu, Xiaohua Meng, Duo Zhang, Di Zhou, Yonghong Xiao, Beiwen Zheng

**Affiliations:** a State Key Laboratory for Diagnosis and Treatment of Infectious Diseases, National Clinical Research Center for Infectious Diseases, Collaborative Innovation Center for Diagnosis and Treatment of Infectious Diseases, the First Affiliated Hospital, Zhejiang University School of Medicine, Hangzhou, China; b Yuhang Institute of Medical Science Innovation and Transformation, Hangzhou, China; c Laboratory of Animal Research Center, the First Affiliated Hospital, Zhejiang University School of Medicine, Hangzhou, China; d The School of Basic Medicine, Beihua University, Jilin, China; e Department of Structure and Morphology, Jinan Microecological Biomedicine Shandong Laboratory, Jinan, China; f Research Units of Infectious Diseases and Microecology, Chinese Academy of Medical Sciences, Beijing, China

**Keywords:** *Elizabethkingia anophelis*, Eravacycline, Omadacycline, Blood infection, Antimicrobial efficacy

## Abstract

**Introduction:**

*Elizabethkingia anophelis* has emerged as a formidable pathogen causing severe, life-threatening human infections. Treatment remains challenging due to its intrinsic multidrug resistance.

**Objectives and methods:**

This study presents the first evaluation of eravacycline and omadacycline efficacy against *E. anophelis in vitro* and *in vivo*. Susceptibility testing of 197 clinical isolates employed broth microdilution. *Galleria mellonella* larval infection and murine bacteremia models assessed therapeutic efficacy.

**Results:**

Minimum inhibitory concentration (MIC) analyses demonstrated potent *in vitro* activity against clinical isolates, with MICs as low as 0.06 mg/L and 0.125 mg/L, respectively. In the *G. mellonella* model, both agents significantly delayed larval mortality (P < 0.01) and improved health indices. Murine studies showed substantial reductions in bacterial burden versus saline controls, alongside significant anti-inflammatory and organ-protective effects. Eravacycline achieved complete bacterial eradication in hepatic, splenic, and pulmonary tissues.

**Conclusion:**

These findings substantiate the robust antimicrobial efficacy of eravacycline and omadacycline against *E. anophelis*, offering valuable insights for optimizing clinical management. They provide compelling proof-of-concept that fluorocyclines eradicate multidrug-resistant *E. anophelis in vitro* and *in vivo*, strongly supporting clinical trial investigation.

## Highlights

• Eravacycline and omadacycline demonstrate potent efficacy against *E. anophelis*, guiding optimized therapy.
• Both agents exhibit potent in vitro activity against *E. anophelis* (MICs: 0.06/0.125 mg/L).
• Eravacycline and omadacycline significantly delayed *G. mellonella* mortality (P<0.01).
• Murine studies confirm potent anti-infective, anti-inflammatory, and organ-protective efficacy of both antibiotics.

## Graphical abstract

**Figure:**
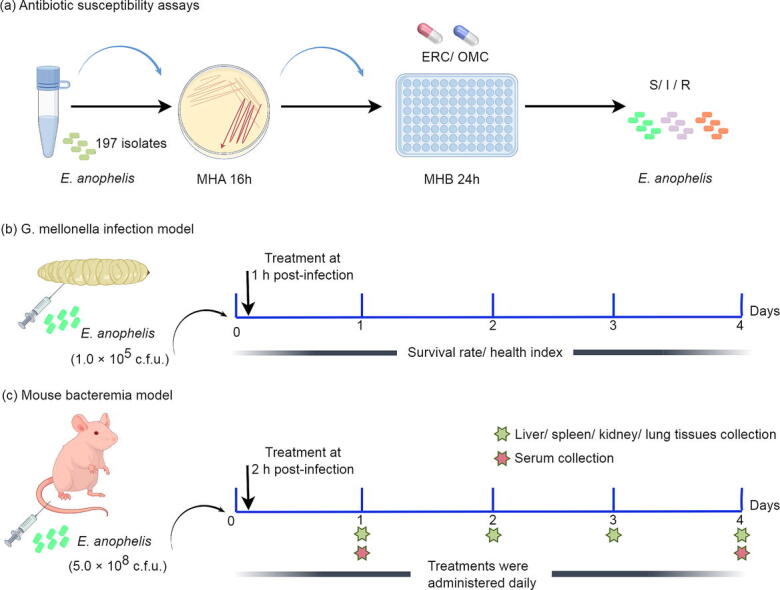

## Introduction

The genus *Elizabethkingia* comprises aerobic, oxidase-positive, non-fermenting, non-motile Gram-negative bacilli ubiquitous in aqueous and terrestrial environments [1,2]. Initially classified as *Flavobacterium* in 1959 by Elizabeth O. King following its identification in infant meningitis cases [3], it was reclassified as *Chryseobacterium* in 1994[4], and subsequently assigned to the novel genus *Elizabethkingia* in 2005 [5]. This genus currently encompasses seven species: *Elizabethkingia anophelis*, *E. argenteiflava*, *E. bruuniana*, *E. meningoseptica*, *E. miricola*, *E. occulta*, and *E. ursingii*. (https://lpsn.dsmz.de/genus/Elizabethkingia). *E. anophelis* has recently emerged as an opportunistic pathogen causing life-threatening infections in immunocompromised individuals, older adults, and neonates [2,6]. A Substantial evidence implicates *E. anophelis* in diverse opportunistic infections including meningitis, pneumonia, bacteremia, endophthalmitis, and urinary tract infections [[7], [8], [9], [10], [11]]. Global outbreaks have occurred, documented in Singapore, the United States, South Korea, France, and Taiwan [[12], [13], [14], [15], [16]]. The most significant outbreak occurred in the United States (November 2015–March 2016), with 57 confirmed cases (56 in Wisconsin, one in Michigan) and 31.6 % mortality (18 fatalities), prompting formal notification to the WHO on April 11, 2016 (https://www.who.int/emergencies/disease-outbreak-news/item/21-april-2016-elizabethkingia-usa-en).

Antimicrobial resistance is a leading global health threat, with emerging multidrug-resistant (MDR) “superbugs” representing a critical public health concern [17]. *E. anophelis* infections pose dual therapeutic challenges: high mortality (24–60 %) [18] and intrinsic multidrug resistance. Clinicians face significant therapeutic challenges due to its broad *in vitro* resistance to multiple antimicrobial classes. Originating from environmental reservoirs, *E. anophelis* exhibits resistance to carbapenems, most β-lactams, β-lactam/β-lactamase inhibitor combinations, aminoglycosides, polymyxins, and frequent resistance to tetracyclines, chloramphenicol, and fluoroquinolones [6,19,20]. While minocycline remains FDA-approved and clinically used for various infections, its applicability to invasive *E. anophelis* requires cautious interpretation and supporting evidence [20,21]. Trimethoprim-sulfamethoxazole (TMP-SMZ) shows variable efficacy depending on infection source [22,23]. Genomic heterogeneity further complicates treatment, as *E. anophelis* harbors chromosomal resistance genes (e.g., *bla*B, *bla*CME, and *bla*GOB) conferring β-lactam resistance[24], alongside virulence factors like lipopolysaccharide, type IV pili, oxidative stress response genes, capsule, and siderophores[25]. Strain variations in antibiotic resistance and pathogenicity arise from diverse isolation sources and evolutionary diversification [26], underscoring the need for comprehensive *in vitro* and *in vivo* antimicrobial efficacy evaluation to identify viable clinical candidates.

Antimicrobial resistance is a growing global public health threat requiring comprehensive strategies, including novel antibiotics. Limited understanding of intervention efficacy, both potential and existing, may impact clinical recommendations[27]. Two novel third-generation tetracycline-class antibiotics, eravacycline and omadacycline, recently received US Food and Drug Administration (FDA) approval. Eravacycline exhibits potent broad-spectrum activity, including comprehensive coverage against Gram-negative pathogens and exceptional efficacy against clinically significant Gram-positive organisms like methicillin-resistant Staphylococcus aureus (MRSA) [28]. It also demonstrates robust activity against MDR microorganisms, particularly those producing extended-spectrum β-lactamases or exhibiting carbapenem resistance [28,29]. Although structurally and mechanistically similar to tigecycline, eravacycline shows 2–4 times greater antimicrobial activity against common clinical Gram-positive and Gram-negative aerobic pathogens [30]. Omadacycline, a semisynthetic minocycline derivative, inhibits bacterial protein synthesis by binding the 30S ribosomal subunit. It possesses broad-spectrum activity against diverse aerobic, anaerobic, and atypical bacteria across Gram-positive and Gram-negative classifications [31,32]. Preclinical studies highlight potent *in vivo* efficacy against critical pathogens including *Streptococcus pneumoniae*, *Mycobacteroides abscessus*, methicillin-susceptible *Staphylococcus aureus* (MSSA), and MRSA [31,32]. Consequently, eravacycline and omadacycline are potential treatments for *E. anophelis* infections.

No comprehensive study has yet systematically evaluated the *in vitro* and *in vivo* efficacy of eravacycline and omadacycline against *E. anophelis*. We determined their *in vitro* antibacterial activity using broth microdilution assays. Furthermore, we assessed their *in vivo* therapeutic potential using *Galleria mellonella* larval and murine infection models, enabling comparative evaluation. To our knowledge, this study represents the first comprehensive assessment of these agents against *E. anophelis* using both *in vitro* and *in vivo* models. Our findings demonstrate potent antibacterial activity for both eravacycline and omadacycline against *E. anophelis*, providing critical insights for clinical management, particularly in bloodstream infections and multi-organ dysfunction cases.

## Methods and materials

### Antibiotic susceptibility assays: MIC

All 197 *E. anophelis* strains analyzed were clinical isolates, as described previously [26], collected between January 2010 and April 2019. The isolates originated from Anhui (three), Fujian (two), Hubei (one), Inner Mongolia (one), and predominantly Zhejiang, China (the remainder). Specimen sources included: sputum (138 isolates), blood (25), abdominal fluid (7), cerebrospinal fluid (5), and bronchoalveolar lavage fluid (5). Samples were primarily obtained from patients in intensive care, hematology, infectious diseases, and surgical wards.

Minimum inhibitory concentrations (MICs) for eravacycline and omadacycline were determined by broth microdilution in cation-adjusted Mueller-Hinton broth following Clinical and Laboratory Standards Institute (CLSI) guidelines [33]. Eravacycline and omadacycline were obtained from GLPBIO (Montclair, USA). Tested concentrations, prepared in two-fold serial dilutions, ranged from 0.015–32 mg/L for eravacycline and 0.03–64 mg/L for omadacycline. The MIC was defined as the lowest drug concentration inhibiting visible bacterial growth after 24 h of aerobic incubation at 37 °C. Due to the absence of established CLSI or EUCAST interpretive criteria (MIC breakpoints) for tigecycline, eravacycline, or omadacycline against *E. anophelis*, results were evaluated using FDA-designated breakpoints: eravacycline susceptibility for *Enterobacteriaceae* (≤0.5 mg/L) and omadacycline thresholds (susceptible: ≤4 mg/L; intermediate: 8 mg/L; resistant: ≥16 mg/L) [34,35]. Quality control was performed using *Escherichia coli* ATCC 25922 and *S. aureus* ATCC 29213 with each batch.

### *G. mellonella* (wax moth) infection model

The therapeutic efficacy of eravacycline and omadacycline was evaluated using a *Galleria mellonella* infection model, following established protocols [21,36]. Larvae (250–350 mg) were used throughout. Caterpillars (Tianjin Huiyude Biotech Company) were randomly assigned to four groups (n = 10/group) and inoculated in the left posterior proleg with 20 µL of *E. anophelis* SKLX037417 suspension (1.0 × 10^5^ CFU). One hour post-infection, treatments were administered via the right posterior proleg: sterile physiological saline solution (SPSS), eravacycline (50 mg/kg), or omadacycline (50 mg/kg) [21]. A control group injected only with SPSS assessed procedure-related mortality. Injected larvae were incubated at 37 °C in sterile containers. Survival rates and health index scores were recorded every 12 h for 96 h. Each treatment group contained ten larvae, with experiments independently replicated in triplicate. Final results represent mean survival percentages from aggregated trial data.

### Mouse infection models /murine bacteremia models

The murine bacteremia model was adapted from established literature[37]. Seven-week-old female BALB/c mice were randomly assigned to treatment or control cohorts. Before experiments, mice acclimated for one week in a controlled vivarium under a 12-hour light/dark cycle. For bacteremia induction, mice (n = 12 per group) received an intravenous tail vein injection of *E. anophelis* SKLX037417 suspension (5.0 × 10^8^ CFU) [37]. This dose, determined in preliminary experiments, optimally induced non-lethal bloodstream infection. Two hours post-inoculation, treatments were administered via daily intraperitoneal injection: SPSS (vehicle control), eravacycline (20 mg/kg), or omadacycline (20 mg/kg). Three mice per cohort were euthanized at 24-hour intervals post-infection. Organs (liver, spleen, kidney, lung) were aseptically excised and homogenized in sterile SPSS for bacterial quantification and histopathology [37]. Tissue homogenates underwent serial 10-fold dilution and plating on Mueller-Hinton agar (MHA; Oxoid, UK) supplemented with 16 mg/L meropenem for enumeration. This concentration was used because SKLX037417 exhibits high meropenem resistance [20] and grows robustly on MHA containing 16 mg/L meropenem (Fig. S1).

For histology, tissue specimens were fixed in 4 % formaldehyde, dehydrated through graded ethanol, cleared in xylene, and paraffin-embedded. Sections (4–6 μm) were cut using a Leica™ microtome and stained with hematoxylin and eosin (H&E) [38]. Slides were analyzed using a 3DHISTECH light microscope (Pannoramic MIDI, Hungary) and SlideViewer Standard Software.

To assess immune responses, serum samples were collected from infected mice. Levels of C-reactive protein (CRP), interleukin-6 (IL-6), and tumor necrosis factor-alpha (TNF-α) were quantified using a commercial ELISA kit according to the manufacturer’s protocols (Ruisaiqi Biotechnology, Wuhan, China). Primary materials and reagents are listed in Table S1.

### Statistical analyses

Statistical analyses were performed using GraphPad Prism 6.0 software. Survival differences were assessed using Kaplan-Meier curves with log-rank (Mantel-Cox) testing. Student’s *t*-test was used for normally distributed data comparisons. Data are presented as mean ± SD, with statistical significance set at P < 0.05.

### Ethics statement

This study complied with the Helsinki Declaration. All animal experiments followed ethical policies and procedures approved by the institutional ethics committee (Approval no. 20201129).

## Results

### Eravacycline and omadacycline are bactericidal *in vitro*

Susceptibility testing of 197 *E. anophelis* clinical isolates revealed high susceptibility to eravacycline (Fig. S2 and Table S2). The lowest eravacycline MIC was 0.06 mg/L, with MICs ≤ 0.5 mg/L for 175 isolates, 1 mg/L for 19, and 2 mg/L for 3. For omadacycline, all isolates were inhibited at ≤ 4 mg/L (100 % susceptibility), with only 2 % exhibiting MICs > 2 mg/L; the lowest MIC was 0.125 mg/L. These *in vitro* findings indicate both antibiotics exhibit potent therapeutic activity against *E. anophelis*.

### Efficacy to against *E. anophelis* strains in *G. mellonella* larvae

**Fig. 1:**
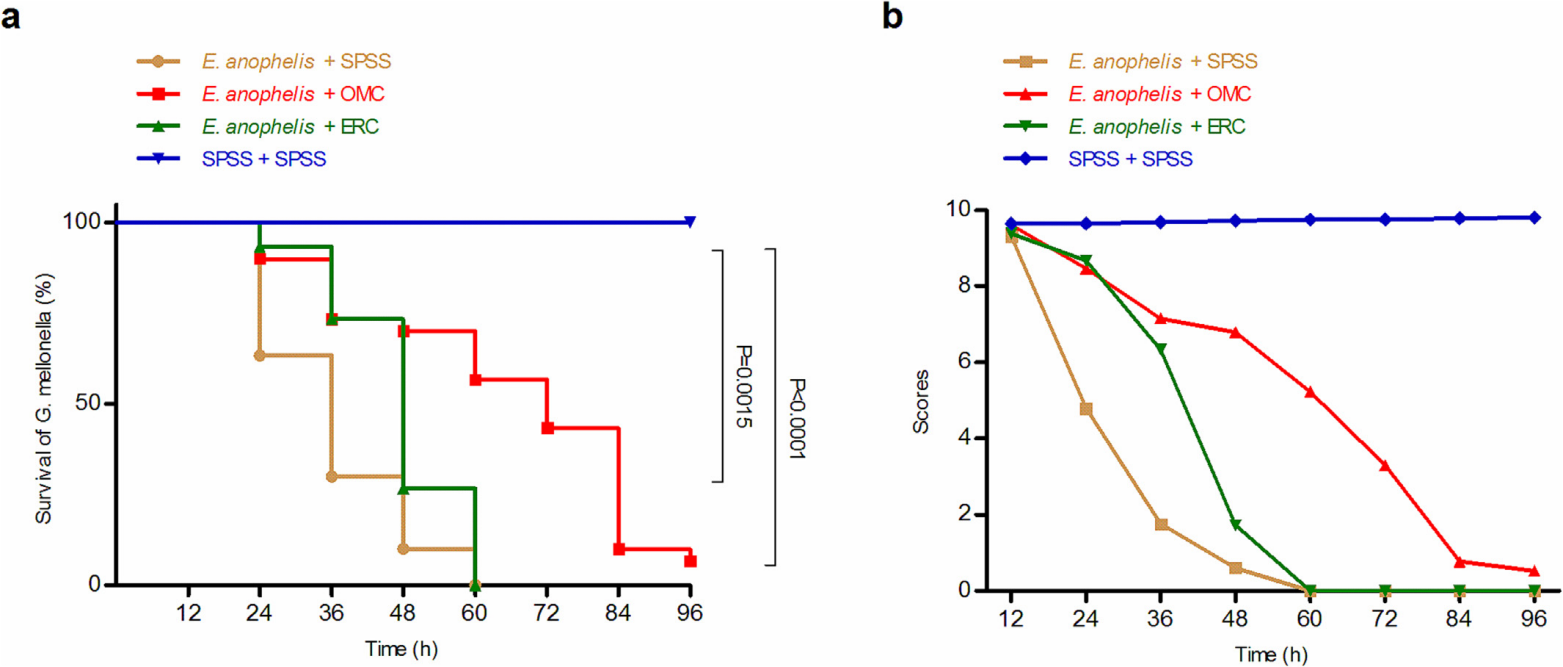
**Efficacy of eravacycline (ERC) and omadacycline (OMC) in *Galleria mellonella* infection assays.** (a) Survival rates of larvae (n = 10 per group) infected with *E. anophelis* (1.0 × 10^5^ c.f.u.) via the left posterior proleg. Treatments—ERC (50 mg/kg), OMC (50 mg/kg), or sterile physiological saline solution (SPSS, control)—were administered via the right posterior proleg 1 h post-infection. (b) Comparative larval health index scores (ERC/OMC vs. control). Health status was evaluated using a standardized Health Index Scoring System based on motility, cocoon formation, melanization, and survival. Data represent means from three independent experiments.

Previous studies indicate that antimicrobial therapy effectively reduces lethal *G. mellonella* infections [39]. To evaluate the suitability of the *G. mellonella*–*E. anophelis* model for assessing *in vivo* antibacterial efficacy, larvae infected with *E. anophelis* received a single dose of eravacycline (50 mg/kg), omadacycline (50 mg/kg), or SPSS. As shown in Fig. 1a, antibiotics demonstrating *in vitro* activity against *E. anophelis* significantly improved survival in lethally infected larvae (P < 0.01). By day 3 post-infection, eravacycline-treated larvae exhibited markedly increased survival rates (P = 0.0015). Similarly, omadacycline administration significantly enhanced 4-day survival rates (P < 0.0001). Kaplan-Meier analysis revealed distinct survival trajectories among eravacycline-, omadacycline-, and SPSS-treated groups post-inoculation. By day 4, omadacycline continued to demonstrate significantly higher survival, indicating a more pronounced therapeutic effect (Fig. 1a). Furthermore, larval health indices were substantially higher in antibiotic-treated groups compared to SPSS controls (Fig. 1b), highlighting their clinical relevance.

### Antibacterial efficacy in the BALB/c mice- *E. anophelis* infection model

**Fig. 2:**
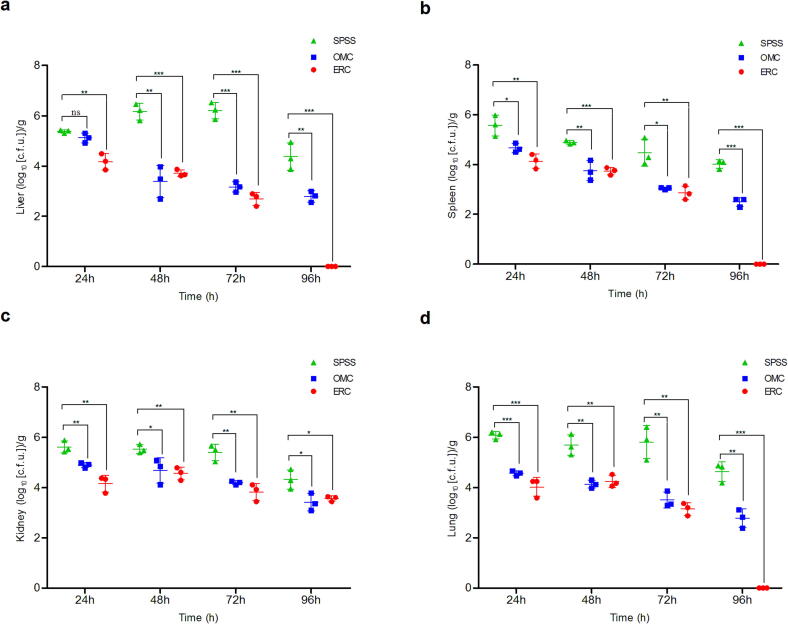
**Bacterial burden in a murine bacteremia model.** Tissue bacterial loads (log_10_ [c.f.u.]/g) in the liver (a), spleen (b), kidney (c), and lungs (d) of mice infected with *E. anophelis* (5.0 × 10^8^ c.f.u.) were quantified at 24, 48, 72, and 96 h post-infection after daily intraperitoneal treatment with ERC (20 mg/kg), OMC (20 mg/kg), or SPSS. ERC and OMC significantly reduced bacterial loads. Data are expressed as means ± standard deviation (SD). Asterisks indicate significant differences (ns: non-significant; * P < 0.05; ** P < 0.01, *** P < 0.001).

Given their promising synergistic potential, we evaluated eravacycline and omadacycline efficacy in murine models. Bacterial burden in liver, spleen, kidney, and lung was assessed post- *E. anophelis* inoculation. One day post-injection, eravacycline-and omadacycline-treated groups exhibited markedly reduced mean bacterial colony counts versus controls, demonstrating robust *in vivo* antibacterial activity (Fig. 2, S3). To evaluate sustained anti-infective capacity, tissue colonization was monitored longitudinally over four days. Eravacycline induced significant bacterial load reductions in hepatic, splenic, and pulmonary tissues relative to the SPSS group, achieving complete microbial eradication (culture-negative) by day 4. Although renal clearance remained incomplete, eravacycline substantially decreased colony counts. Similarly, omadacycline significantly diminished bacterial levels across all organs by the endpoint versus controls (Fig. 2, S3).

**Fig. 3:**
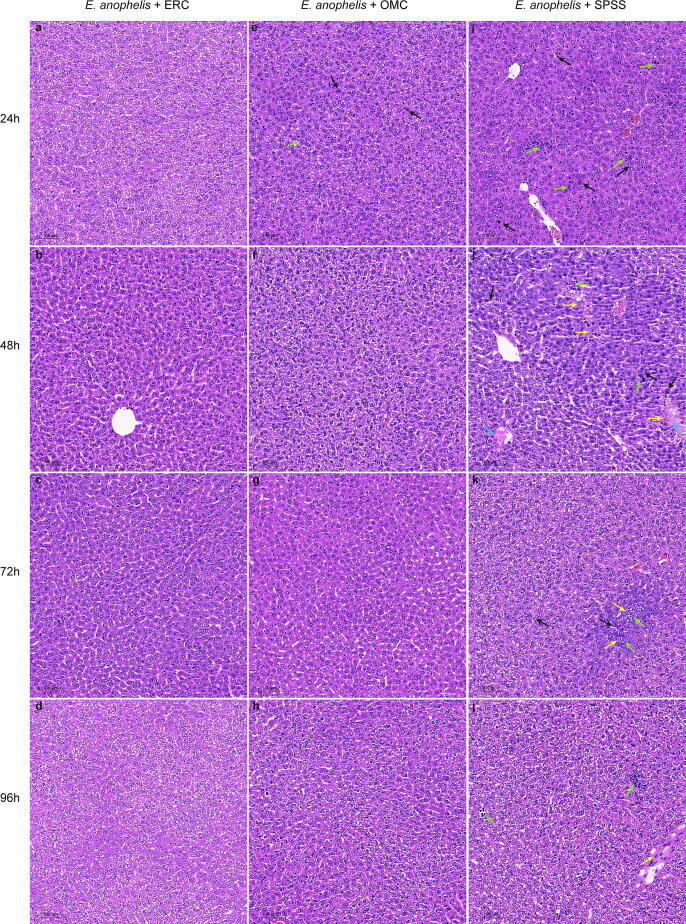
**Histopathologic examination of liver tissues.** Representative photomicrographs of hepatic histology in control (SPSS-injected) and treatment groups (20 mg/kg ERC- or 20 mg/kg OMC-administered mice) assessed via H&E (hematoxylin and eosin) staining. Baseline magnification: 100×; scale bar: 100 μm (lower panel). (a–d): ERC daily intraperitoneal administration (i.p.); (e–h): OMC daily, i.p.; (i–l): Vehicle control (SPSS). ERC and OMC groups showed attenuated pathology vs. control: hemorrhage (black arrow), inflammatory infiltration (green arrow), cytoplasmic vacuolization (blue arrow), and hepatocyte necrosis (yellow arrow).

**Fig. 4:**
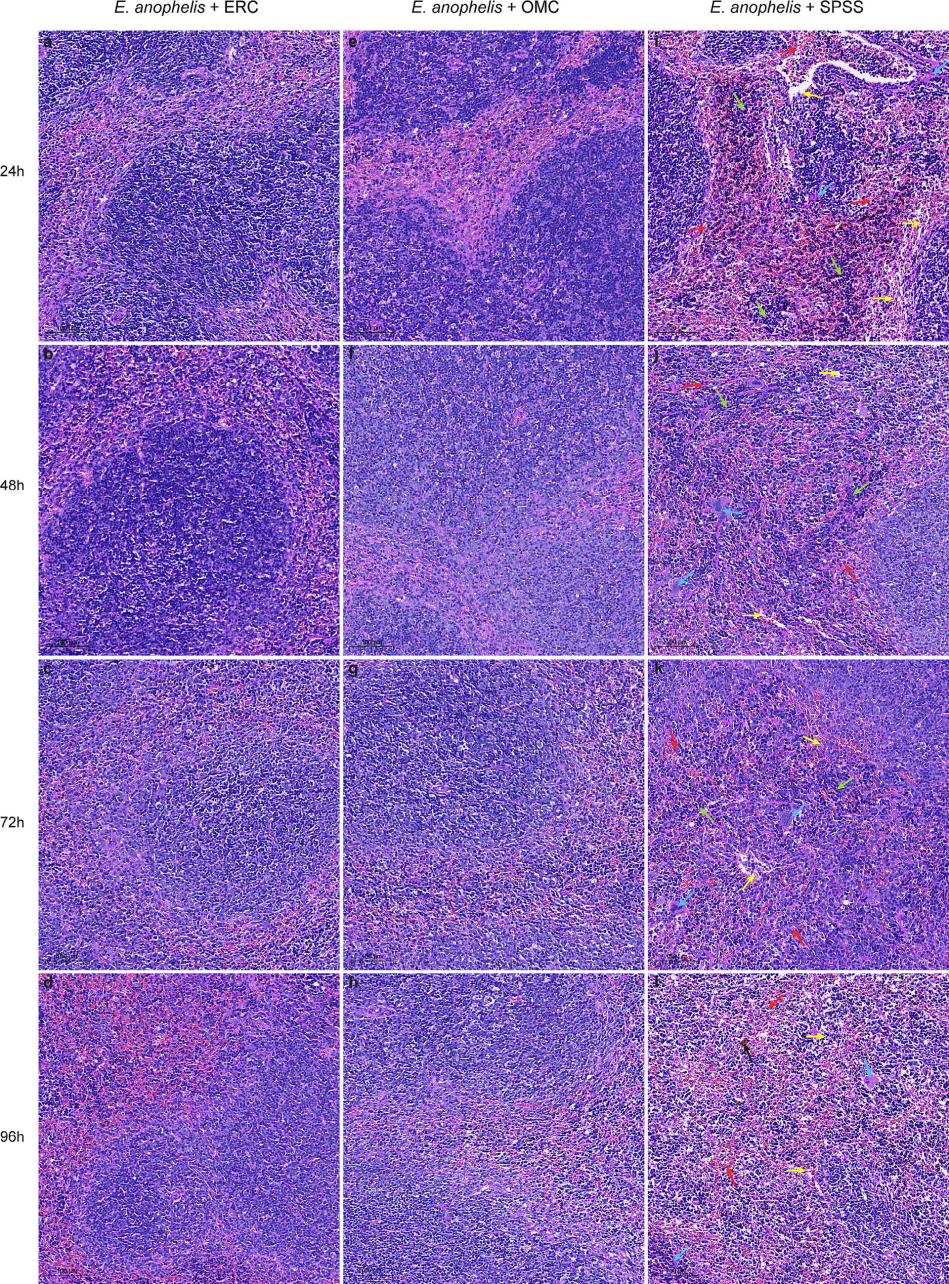
**Splenic histopathology in bacteremic mice infected with *E. anophelis* (5.0 × 10^8^ c.f.u.).** Representative sections (100 × magnification; scale bar: 100 μm). (a–d): ERC (20 mg/kg daily, i.p.); (e–h): OMC (20 mg/kg daily, i.p.); (i–l): SPSS control. Relative to control, ERC and OMC attenuated red/white pulp depletion (red arrow), inflammatory infiltration (green arrow), hyaline lesions (blue arrow), hemorrhage (black arrow), and necrosis (yellow arrow).

Histological analyses of multiple organs validated anti-infection efficacy. Eravacycline- or omadacycline-treated mice showed markedly improved health indices and attenuated pathological alterations. Diseased mice exhibited pronounced lesions, including hyperemia, cellular degeneration, necrosis, and multi-organ inflammation (Fig. 3, Fig. 4, S4, S5). Tissue damage severity varied among eravacycline, omadacycline, and SPSS groups. SPSS hepatic sections revealed significant pathology: hepatocyte swelling, hemorrhage, hyaline and vacuolar degeneration, necrotic foci, and inflammatory infiltration (Fig. 3). Splenic architecture displayed severe disruption, with loss of red/white pulp, inflammatory infiltration, hyaline lesions, and necrosis (Fig. 4). Renal tissues showed vacuolar/hyaline degeneration, cellular necrosis, interstitial congestion, and inflammation (Fig. S4). Pulmonary histology indicated interstitial thickening, alveolar exudate, hyperemia, and lymphocytic inflammation (Fig. S5). In contrast, eravacycline and omadacycline groups exhibited minimal hemorrhage, inflammation, degeneration, or necrosis across all organs.

**Fig. 5:**
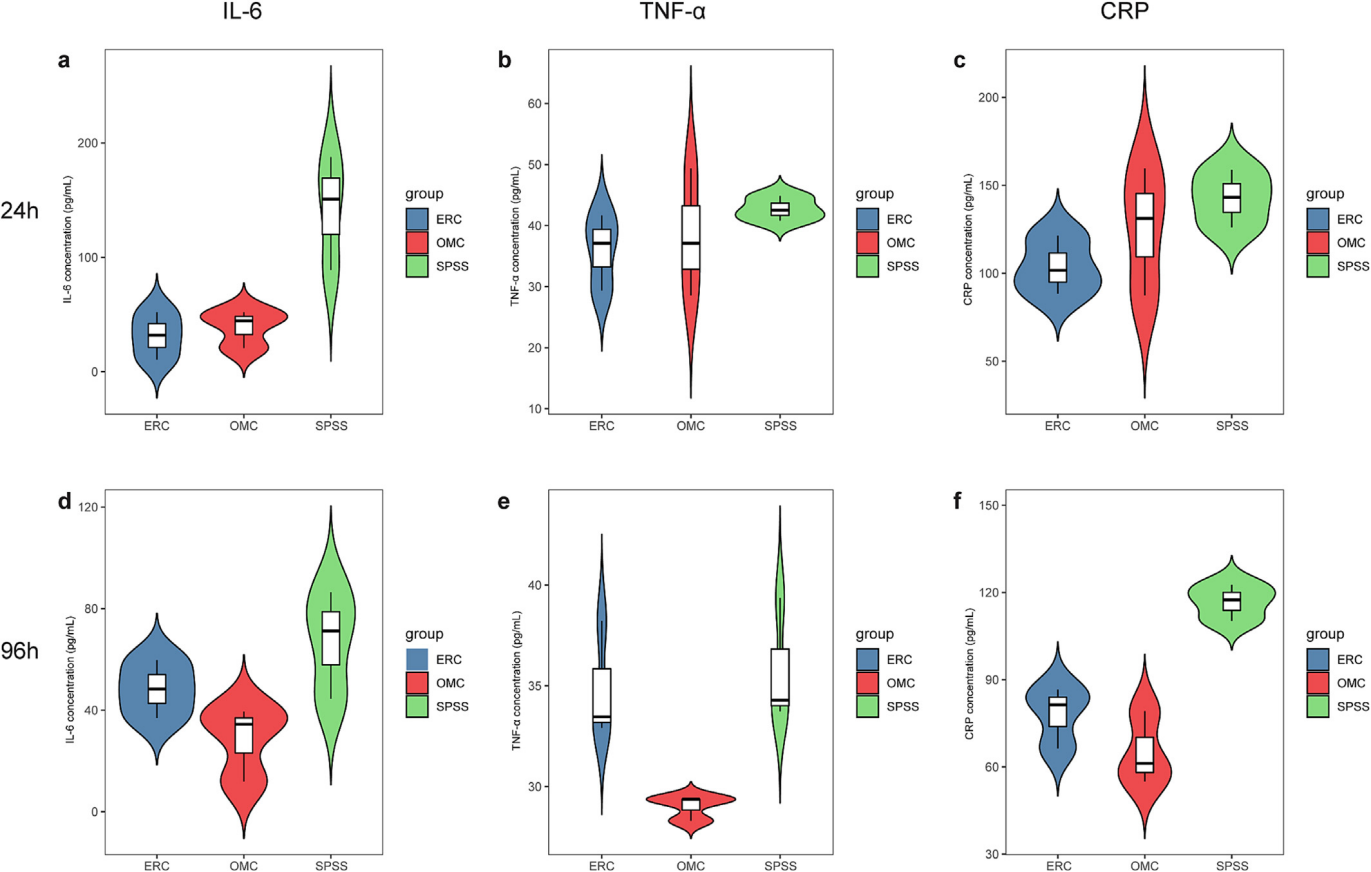
**Serum inflammatory factors examination**. Serum levels of interleukin-6 (IL-6; a, d), tumor necrosis factor-α (TNF-α; b, e), and C-reactive protein (CRP; c, f) in mice across treatment groups. Concentrations were quantified by ELISA at 24 h and 96 h post-infection with *E. anophelis*.

ELISA analysis of serum cytokines IL-6, TNF-α, and CRP showed significantly lower levels in eravacycline and omadacycline groups versus SPSS cohorts (Fig. 5). SPSS groups had markedly elevated cytokine concentrations. Conversely, eravacycline- or omadacycline-treated mice showed only modest post-infection cytokine increases compared to SPSS-treated mice (Fig. 5). These findings align with quantitative tissue inflammation data, corroborating the potent anti-infection efficacy of both agents.

## Discussion

*E. anophelis*, an increasingly concerning opportunistic pathogen, poses a critical public health threat due to its high mortality rates and propensity for outbreaks. As documented, *E. anophelis* exhibits resistance to nearly all beta-lactams, including carbapenems, and most commonly used antimicrobials [2,19,20]. Given the prevalence of multidrug resistance and the lack of established clinical therapies, novel antibiotics and treatment strategies are urgently required. This study evaluates the *in vitro* and *in vivo* efficacy of third-generation tetracyclines, eravacycline and omadacycline, against *E. anophelis*. To our knowledge, it provides the first comprehensive evaluation of both agents against *E. anophelis* in both *in vitro* and animal models. Our findings confirm the potent antibacterial activity of eravacycline and omadacycline, offering critical guidance for treating challenging clinical cases such as bloodstream infections and multi-organ dysfunction.

In the present study, *E. anophelis* isolates exhibited high susceptibility to eravacycline and omadacycline. For MIC assays, FDA breakpoints for *Enterobacteriaceae* were applied to *E. anophelis*, which belongs to *Flavobacteriaceae*, not *Enterobacteriaceae*; this taxonomic disparity may compromise susceptibility categorization validity. This limitation primarily arises from the absence of CLSI and EUCAST MIC breakpoints for tigecycline, omadacycline, and eravacycline against *E. anophelis*. Although inevitable, surrogate breakpoints remain reliable, as prior studies demonstrate that MIC testing of tigecycline, eravacycline, and omadacycline against *E. anophelis* routinely employs FDA-defined *Enterobacteriaceae* standards[35,40,41].

Our study demonstrates promising *in vitro* and *in vivo* antimicrobial activity of eravacycline and omadacycline against MDR *E. anophelis*. Although both agents are active, they exhibit significant efficacy differences in a murine bacteremia model. Statistical analysis of tissue bacterial counts confirmed that eravacycline's antibacterial activity and restorative effects in the liver and spleen are significantly superior to omadacycline (Fig. 2). Colony counts were significantly lower in the eravacycline group compared to the omadacycline group at four time points versus the control group, with generally smaller p-values. Notably, liver and spleen tissues in the eravacycline group were culture-negative at 96 h. Cytokine analysis corroborated these findings (Fig. 5). Serum analysis revealed no significant difference in IL-6 and TNF-α concentrations between treatment groups at 24 h; CRP concentration was higher in the eravacycline group. By 96 h, however, serum IL-6, TNF-α, and CRP concentrations were all significantly lower in the eravacycline group.

In lung tissue, bacterial clearance did not differ significantly between eravacycline and omadacycline during the first 72 h. Eravacycline demonstrated superior clearance by 96 h, achieving complete eradication. In contrast, liver tissue showed no statistically significant difference in bacterial load between the control and omadacycline groups at 24 h, although omadacycline reduced the load over the subsequent 72 h. Pathological examination confirmed tissue repair (Fig. 3). This phenomenon may relate to PK/PD parameters and warrants further investigation. Both eravacycline and omadacycline exhibited significantly lower bacterial clearance efficacy in renal tissue compared to liver, spleen, and lung tissues. This reduced efficacy may stem from lower renal drug concentrations, necessitating further research.

Although prior studies indicate *E. anophelis* susceptibility to various antimicrobials, it exhibits resistance to numerous antibiotics, complicating effective treatment guidance. Consistent with our findings, minocycline is highlighted as a viable option due to observed susceptibility [[20], [21], [22],^42^]. However, its utility may be limited in regions like China, where only oral formulations are available [23]. Notably, *in vivo* studies reveal spontaneous mutations associated with reduced minocycline susceptibility, contradicting initial reports and underscoring emerging resistance risks during therapy [21]. While levofloxacin and ciprofloxacin are potential treatments, highly variable fluoroquinolone resistance rates compromise their efficacy, making them suboptimal [16,19,42,43]. Although piperacillin-tazobactam and TMP-SMX have historical success, efficacy varies across infection sources [22,23]. Our prior research demonstrated high resistance rates for these agents: 29 % for piperacillin-tazobactam and 99 % for TMP-SMX [20]. Additionally, TMP-SMX exhibits substantial inter-case variability in minimum inhibitory concentrations (MICs), compounded by frequent susceptibility testing discrepancies [35,40,44]. Due to inconsistent MIC findings and unreproducible efficacy, TMP-SMX should not be a cornerstone therapy for invasive *E. anophelis* infections.

Eravacycline and omadacycline demonstrate broad-spectrum efficacy against Gram-positive and Gram-negative bacteria. Nevertheless, increasing fluorocycline resistance mechanisms have emerged. For instance, a branched-chain amino acid transport system II carrier protein can modulate eravacycline and omadacycline susceptibility in *S. aureus* [45,46]. Findings suggest these antibiotics are suboptimal for ESBL-producing *Klebsiella pneumoniae* harboring *bla*TEM, as *bla*TEM may co-express tetracycline resistance determinants [34]. Mutations within resistance determinant domains also contribute significantly; lon gene mutations enhance *K. pneumoniae* eravacycline resistance [47]. Eravacycline resistance in enterococci may arise from combined mechanisms (*rpsJ* mutations with *tet* genes) [48]. Furthermore, a novel mutated *tet(L)* efflux pump conferring eravacycline and tigecycline resistance has been identified in *Staphylococcus* species [49]. However, our study lacked resistance mechanism characterization. Genomic sequencing of *E. anophelis* and monitoring fluorocycline target mutations are thus essential to guide clinical use of eravacycline and omadacycline.

In natural environments, bacteria secrete outer membrane vesicles (OMVs) transporting essential biomolecules. OMVs facilitate horizontal gene transfer—termed Vesiduction—of resistance genes, genetic elements, DNA, RNA, and proteins [50,51]. Enriched with virulence factors, survival proteins, and carbohydrate-metabolizing enzymes, these nanostructures are promising next-generation vaccine candidates against bacterial pathogens [52]. Recent studies validate imipenem-induced OMVs (iOMVs) as prophylactic and therapeutic agents against *E. anophelis* infections [53]. However, OMVs contain both immunoprotective components and non-protective antigens, complicating the isolation of epitopes eliciting exclusively beneficial immune responses [53,54].

Although this investigation provides critical insights into eravacycline and omadacycline efficacy against *E. anophelis*, several limitations exist. First, only a single *E. anophelis* strain (SKLX037417) was examined due to the assay's laborious and protracted nature, potentially limiting generalizability. Second, antibiotic evaluation was restricted to eravacycline and omadacycline, omitting other potentially efficacious agents (e.g., minocycline, doxycycline, and rifampin), constraining therapeutic inferences. Lastly, while the female BALB/c murine model shares homologous organ systems and immune responses with humans, its physiological simplicity necessitates further studies in diverse mammalian species to confirm translational applicability. Additionally, the murine model sample size was insufficient (n = 12 per group; n = 3 per time-point after sacrifice), limiting robust statistical analysis. These constraints highlight critical avenues for future research.

## Conclusions

The emergence of life-threatening *E. anophelis* infections and their high mortality rates underscore the critical need for developing targeted therapies. Collectively, our findings demonstrate the potent antimicrobial efficacy of eravacycline and omadacycline against *E. anophelis,* particularly in bloodstream infections where their anti-inflammatory properties and protection against organ damage are most evident. These fluorocyclines show promise as candidates for compassionate use protocols and investigator-initiated trials targeting invasive MDR *E. anophelis*. Significantly expanded preclinical validation and rigorous clinical studies are essential to confirm efficacy, optimize dosing, and establish clinical utility against human *E. anophelis* infections.

## Compliance with ethics Requirement

This study was conducted in conformed the Helsinki Declaration, all experiments involving animals were conducted according to the ethical policies and procedures approved by the ethics committee of the First Affiliated Hospital, College of Medicine, Zhejiang University (Approval no. 20201129).

## Declaration of competing interest

The authors declare that they have no known competing financial interests or personal relationships that could have appeared to influence the work reported in this paper.
