# Supplementary material for: Eravacycline and omadacycline Rescue multidrug-resistant Elizabethkingia anophelis: Triple-Model evaluation across in Vitro, Galleria mellonella, and murine bacteremia systems
Source: J Adv Res. 2025 Nov 24;86:757–66. doi: 10.1016/j.jare.2025.11.048 (PMC13453614; doi:10.1016/j.jare.2025.11.048)
Supplement: Supplementary file 1 [file mmc1.pdf]

Supplemental Figures

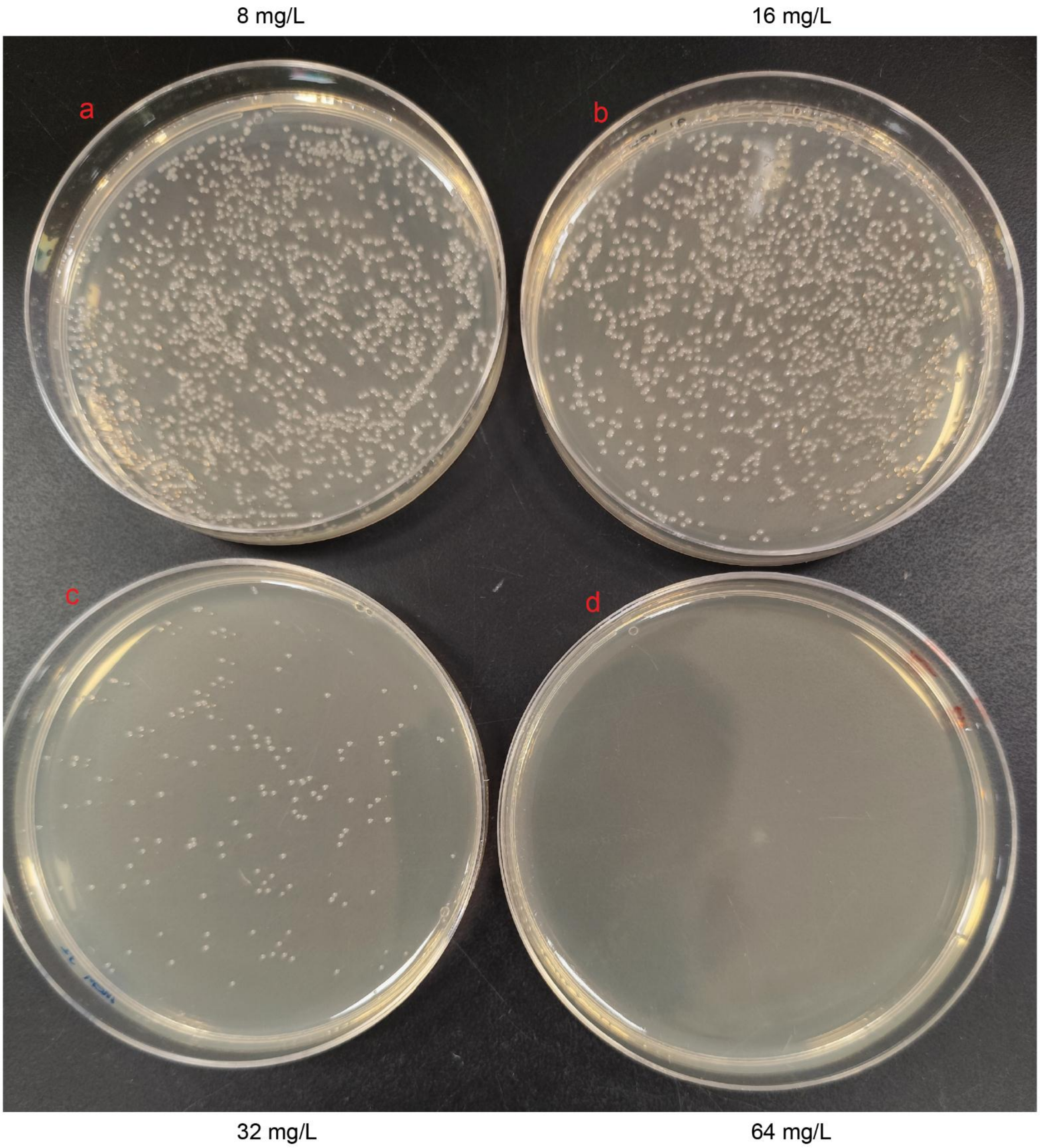

Figure S1. Growth of strain SKLX037417 on meropenem-supplemented medium. Four Mueller Hinton Agar (MHA) plates containing meropenem at concentrations of: a) 8 mg/L, b) 16 mg/L, c) 32 mg/L, and d) 64 mg/L. Plates were inoculated with approximately 1.5×10^{3}c.f.u. (in 100 μL) and incubated at 37 ℃ for 24 hours. Strain SKLX037417 exhibited growth on MHA supplemented with 16 mg/L meropenem.

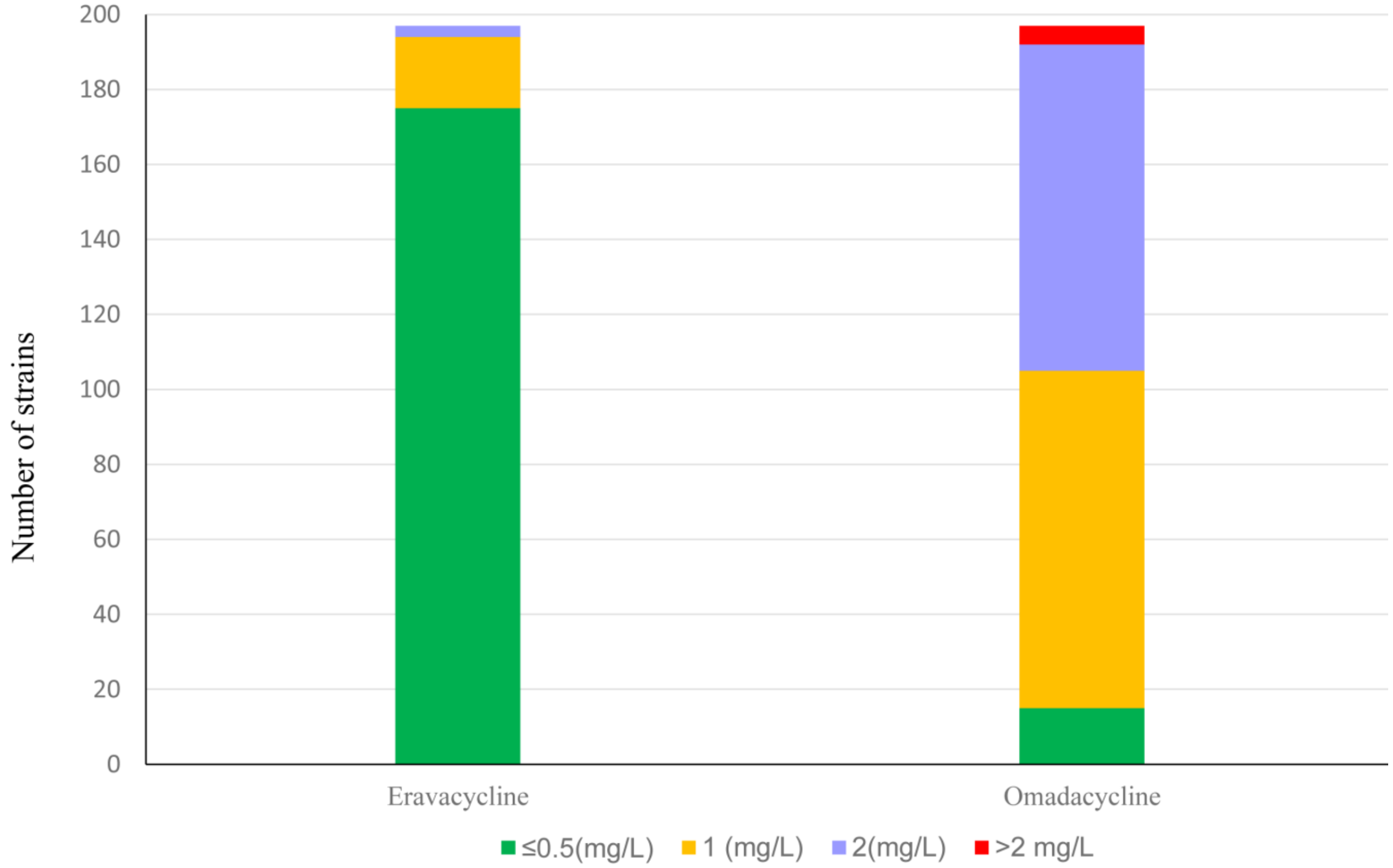

Figure S2. Minimum inhibitory concentration (MIC) distributions for eravacycline and omadacycline across 197 *E. anophelis* isolates. Vertical axis: isolate count. MIC ranges are demarcated by color: green (≤0.5 mg/L), yellow (1 mg/L), purple (2 mg/L), red (>2 mg/L).

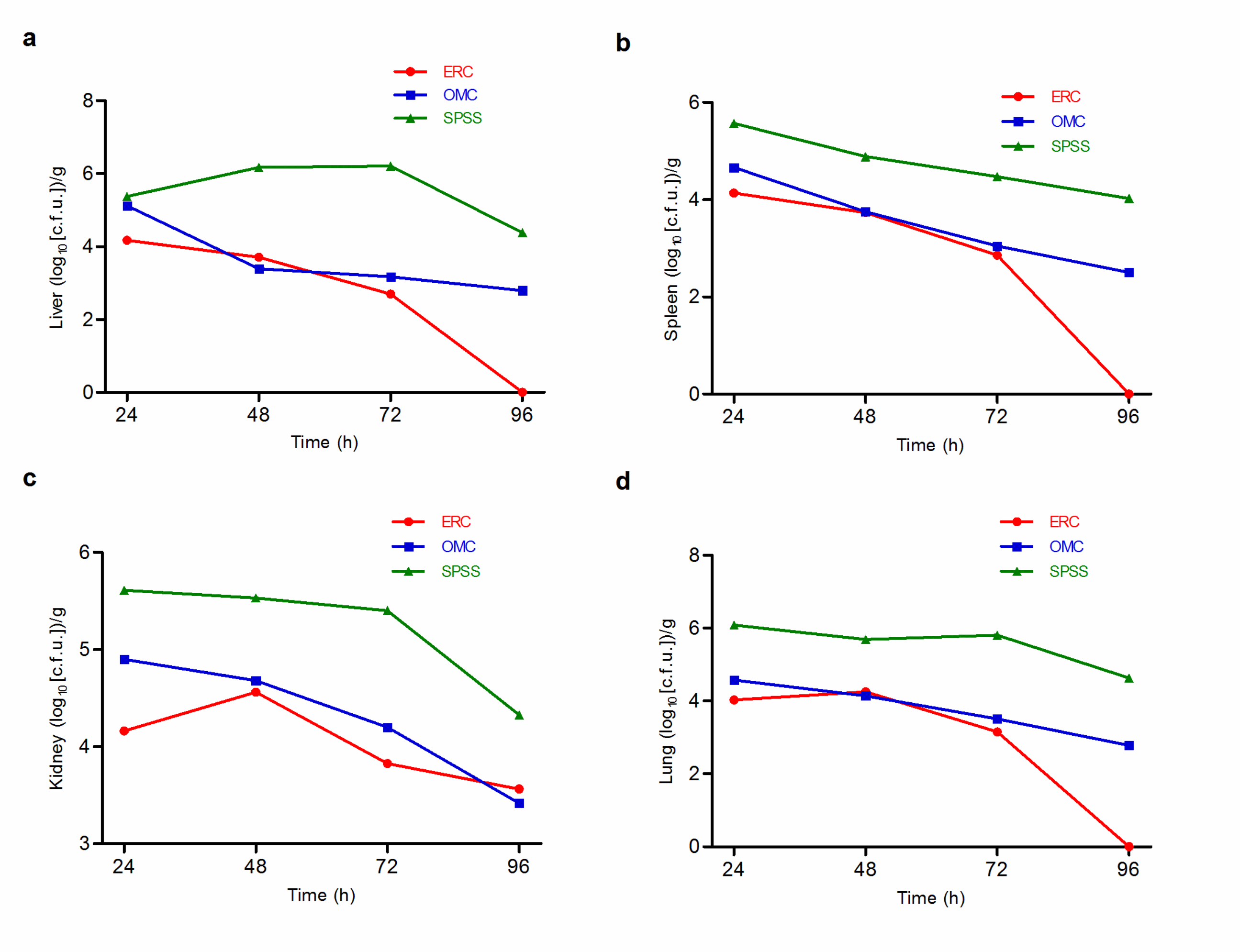

Figure S3. Time-course curves of bacterial counts in a murine bacteremia model. Bacterial loads in the liver (a), spleen (b), kidney (c), and lungs (d) of infected mice 24, 48, 72, and 96 hours post-infection following treatment with eravacycline (ERC), omadacycline (OMC), or sterile physiological saline solution (SPSS). Each point represents the mean bacterial load from three mice. The significant reduction in bacterial load over the 4-day period demonstrates the in vivo antibacterial efficacy of ERC and OMC.

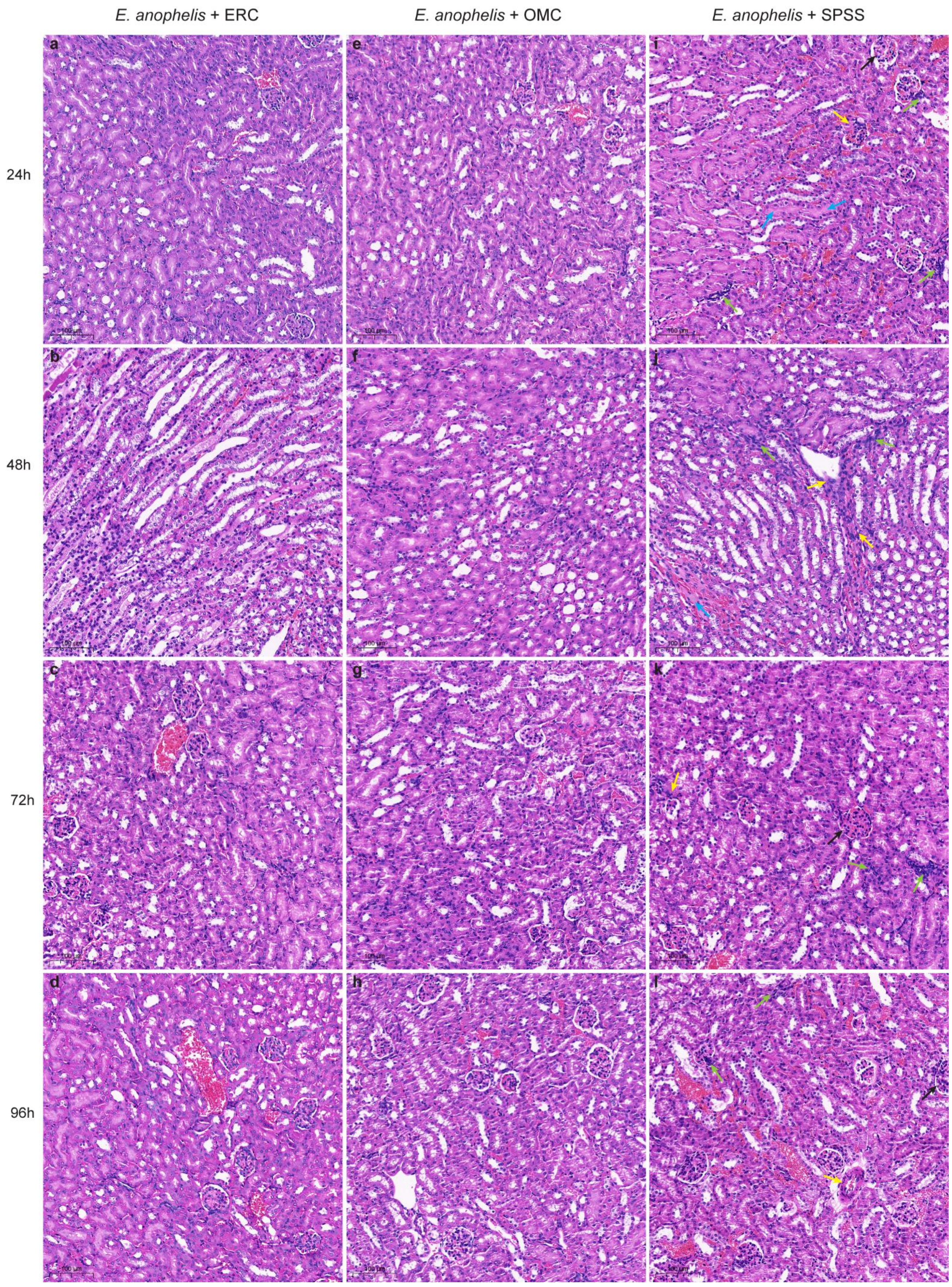

E. anophelis + ERC
E. anophelis + OMC
E. anophelis + SPSS
24h
48h
72h
96h
a
b
c
d
e
f
g
h
i
j
k
l

Figure S4. Histopathological evaluation. Hematoxylin and eosin (H&E)-stained sections of murine renal tissue adjacent to implantation sites at days 1, 2, 3, and 4 post-infection with *E. anophelis*-induced bacteremia. Baseline magnification: 100×. Scale bar: 100 μm (lower left). Panels a–d: Cohort administered 20 mg/kg ERC daily via intraperitoneal injection; Panels e–h: Cohort administered 20 mg/kg OMC daily via intraperitoneal injection; Panels i–l: Control cohort treated with SPSS. Key: Congestion and inflammatory cell infiltration (green arrow), vacuolar and hyaline degeneration (blue arrow), cellular necrosis (yellow arrow), glomerular enlargement (black arrow).

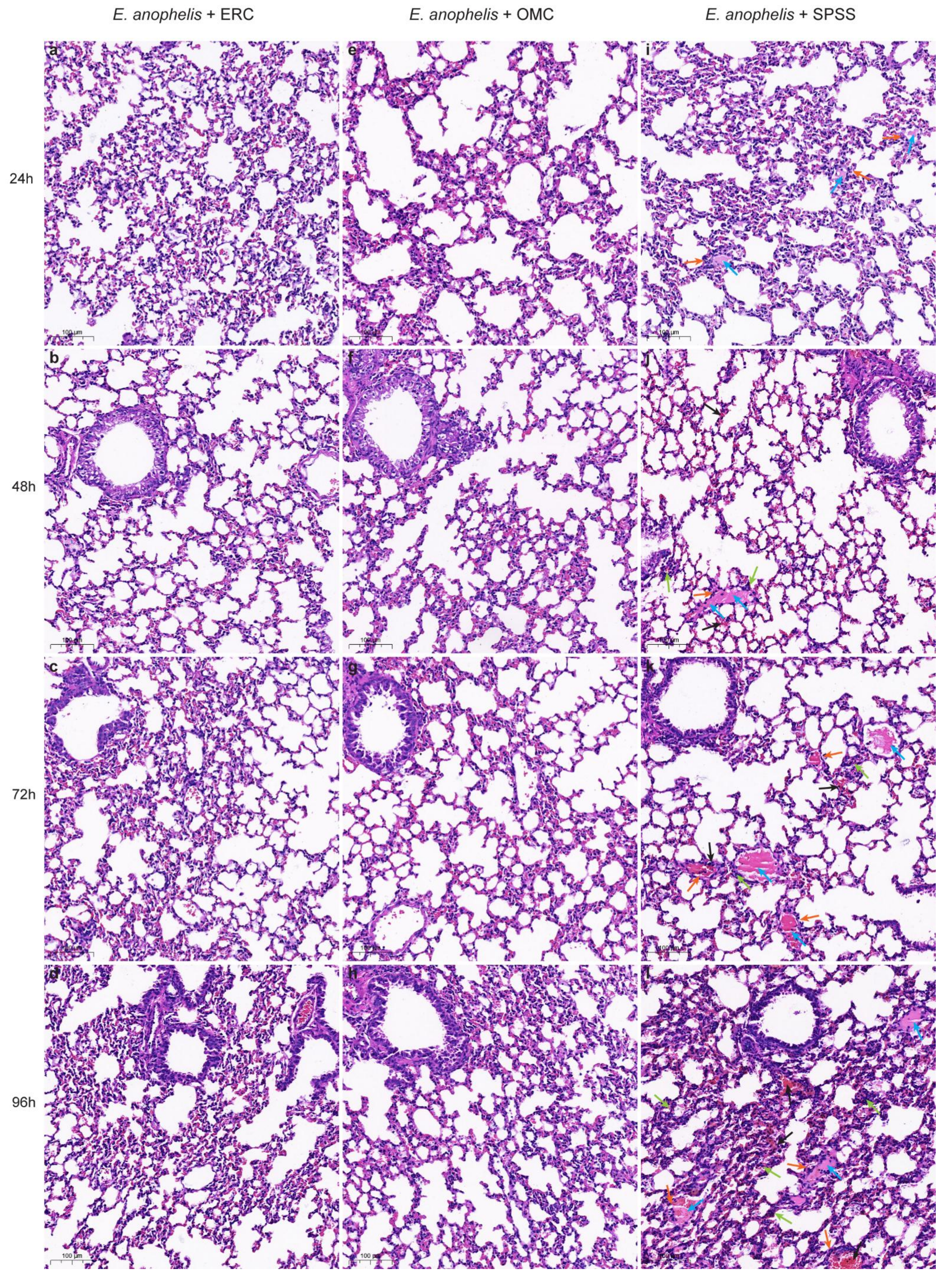

E. anophelis + ERC
E. anophelis + OMC
E. anophelis + SPSS
24h
48h
72h
96h
a
b
c
d
e
f
g
h
i
j
k
l
100 μm

Figure S5: Effects of ERC and OMC on pulmonary inflammatory response in *E. anophelis*-induced bacteremic mice. Original magnification: 100×. Scale bar: 100 μm (shown below). Panels a–d: Cohort administered 20 mg/kg ERC daily via intraperitoneal injection; Panels e–h: Cohort administered 20 mg/kg OMC daily via intraperitoneal injection; Panels i–l: SPSS vehicle control cohort. Key: Hyperaemia (black arrow), lymphocytic inflammation (green arrow), alveolar exudate (blue arrow), alveolar septal thickening (orange arrow).
